# Turning the gaze: Digital patient feedback and the silent pathology of the NHS

**DOI:** 10.1111/1467-9566.13419

**Published:** 2021-12-04

**Authors:** Catherine M. Montgomery, John Powell, Kamal Mahtani, Anne-Marie Boylan

**Affiliations:** 1 Science, Technology and Innovation Studies, School of Social and Political Science, University of Edinburgh, Edinburgh, UK; 2 Centre for Biomedicine, Self and Society, University of Edinburgh, Edinburgh, UK; 3 Nuffield Department of Primary Care Health Sciences, University of Oxford, Oxford, UK

**Keywords:** digital health, Foucault, patient experience, primary care

## Abstract

Online review and rating sites, where patients can leave feedback on their experience of the health-care encounter, are becoming an increasing feature of primary care in the NHS. Previous research has analysed how digital surveillance is re-shaping the clinical gaze, as health-care professionals are subject to increased public monitoring. Here, we draw on an empirical study of 41 GP practice staff to show how the gaze is turning, not simply from the patient to the health-care provider, but additionally to the body politic of the NHS. Drawing on focus group and interview data conducted in five UK practices, we show how discourses of online reviews and ratings are producing new professional subjectivities among health-care professionals and the extent to which the gaze extends not only to individual health-care interactions but to the health-care service writ large. We identify three counter-discourses characterising the evolving ways in which online reviews and ratings are creating new subjects in primary care practices: victimhood, prosumption versus traditional values and taking control. We show how the ways in which staff speak about online feedback are patterned by the social environment in which they work and the constraints of the NHS they encounter on a day-to-day basis.

## Introduction

The rise of consumerism within health care has long been a subject of critical analysis within sociological research, as has more recently the role of patient experience narratives in contributing to this (Lupton, 1997a, 2014). Within these analyses, the changing nature of the doctor–patient relationship has come to the fore, variously focussing on the ascendancy of patient-/person-centred care (Gothill & Armstrong, 1999; May et al., 2006) and the declining authority and professional status of doctors (Armstrong, 1990; Lupton, 1997b). The role of the patient's voice, both within the clinical encounter and beyond (for example, in patient advocacy groups and third sector organisations) has been a defining focus for understanding the micro-politics of power and larger shifts in macrostructural and policy domains (Mazanderani et al., 2020). In this paper, we build on this scholarship to advance an understanding of how digital patient feedback in primary care is changing the nature of surveillance and subjectification in medicine. Against the discursive backdrop of consumer choice and patient empowerment, growing since 1970s, we examine a counter-discourse of disempowerment and demoralisation amongst primary care providers in the UK National Health Service (NHS).

The role of the consumerism movement in healthcare in ushering in a model of ‘patient-centred care’ is well documented (Powell & Boden, 2012), and the labels by which patients have come to figure in policy terms now almost iconic: the ‘resourceful patient’ (Gray, 2002), the ‘autonomous patient’ (Coulter, 2002), the ‘expert patient’ (Shaw & Baker, 2004) and the ‘reflexive patient’ (Adams, 2011). As new digital tools come to feature ever more prominently in health service provision, the figure of the healthcare consumer is morphing into the ‘digital health citizen’ (Powell & Deetjen, 2019). Within this context, attention to the changing figure of the healthcare provider—both *qua* individual and *qua* institution—has been less sustained. While an extensive body of work considers the ways in which patient subjectivities are changing with the advent of digital health and its extended capacities for surveillance (Adams et al., 2017; Dubbeld, 2006; Erikainen et al., 2019; Lupton, 2012), the connection between surveillance and subjectification as it relates to the healthcare provider has not been made.

Online review and rating sites, through which patients can leave feedback on their experience of the healthcare encounter, are positioned in the policy discourse as a tool for achieving patient-centred care (Duschinsky & Paddison, 2018) and are a prime example of the promissory discourse of digital health policy (Henwood & Marent, 2019). They have gathered pace both as a tool in the quality improvement armamentarium and as an object of academic analysis. The literature details what patients think about leaving feedback online (Patel et al., 2016); what healthcare staff think of online patient feedback, how they understand it, and how they respond to it (Baines et al., 2021; Ramsey et al., 2019; Turk et al., 2020); how different kinds of feedback relate to each other (Boylan et al., 2020); and how different kinds of feedback can be classified (Dudhwala et al., 2017). Research conducted to date has conceptualised online patient feedback as a technological extension of traditional feedback and focussed on describing the content of posts, the types of people who leave posts and the types of organisations that do and do not respond. Issues thrown up by the medium have been of central concern, including the ‘anonymity paradox’ (Speed et al., 2016) and user bias and validity (Patel et al., 2015).

A survey of health professionals suggested that doctors in particular have a sceptical view of the value of such feedback, reporting that they rarely encourage it, as they view it as unrepresentative and with limited value for improving the quality of health services (Atherton et al., 2019). They are also sceptical about the platform on which it is obtained, with NHS sanctioned websites (e.g. Care Opinion, NHS Choices) being viewed as providing more potentially transformative feedback than other unregulated sources. Online review platforms in healthcare are of course part of a wider, increasingly ubiquitous social phenomenon of user-generated feedback. As such, the cultural norms around this phenomenon are important to consider. While reviews and ratings in sectors such as travel and retail have been heralded as driving consumer-centred quality improvement, they are also seen as frequently negative, critical or punitive, and the threat of a bad review is now a social media tool in the hands of the contemporary consumer. However, sociological research into this phenomenon specifically within the context of health care suggests that comments and ratings from patients should be seen instead as a means of ‘caring for care’ (Mazanderani et al., 2021). Here, the context of care differentiates healthcare feedback from other forms of service-related reviews: Mazanderani et al. (2021) found that people leaving reviews are in the unique situation of both *depending on* care from the NHS whilst also actively *caring for* it as a symbolic entity.

What remains to be understood, then, is why, in the face of disconfirming evidence, practitioners perceive online feedback to be overwhelmingly negative, and how online feedback is fundamentally changing the social contract between healthcare providers and the public. The latter point suggests that we see online patient feedback not as a new tool, but—following Armstrong, almost 40 years ago—as an artefact of changes in perception, ‘which enable some things to be heard, and not others’ (Armstrong, 1984).

### The patient voice, surveillance and subjectification

To make sense of the changing social relations in primary care in the face of increasing online patient feedback, we turn to Foucauldian analyses of medicine to consider the links between the patient voice, surveillance and subjectification. At the end of the eighteenth century, so Foucault (1975), the medical gaze shifted, from deciphering symptoms on the surface of the body to locating the illness—the lesion—within the hidden interior of the body through medical examination. What the patient said could provide clues, but the elucidation of symptoms by the patient was no longer paramount; rather, what the doctor found inside the patient's body was key. Charting shifts in the role of the patient's voice, Armstrong notes how in the first half of the 20^th^ century, ‘concern with the accuracy and relevance of the patient's own reports crystallised a new object for medical scrutiny in the space previously occupied by intra-corporal pathology. Not only could symptoms be construed as indicators of disease but also as the idiosyncratic expression of some aspect of patienthood’ (Armstrong et al., 2007).

The nature of patienthood had already been the subject of Armstrong's earlier analysis of ‘the patient's view’ (Armstrong, 1984). Presciently, he questioned ‘whether patienthood can exist in spaces other than those traversed by medical perception’:

What then is the patient's view? What is it that the patient says? The problem is one of perception, of the difference between hearing and saying. The patient's view cannot be described or isolated simply as what is said, fundamentally the patient's view is bound up with what is heard. In this sense the patient's view is an artefact of socio-medical perception.

…

Is there a form of experience and expression which escapes the confines of medicalised illness? In part this is an empirical question; but it also raises the question of whether patienthood can exist in spaces other than those traversed by medical perception.

Building on the notion that ‘the patient's view is bound up with what is heard’, in this paper we ask what primary care staff hear when patients report their views online. Conceptually, we are attuned both to the notion that patients can speak for a silent pathology (at both individual and organisational levels) and that changes in their mode of doing so can signal changes to what we understand by patienthood and where it is located. When the patient's view is articulated beyond the clinic, it exists in spaces traversed by medical perception yet not controlled by it. Further, when expression relates not only to ‘medicalised illness’ but to the experience of seeking care, there is potentially a fundamental shift in the relationship between the patient's view, the pathology and medical caregiving.

Adams wrote extensively on this changing relationship in the context of online patient feedback (Adams, 2011, 2013, 2017). Drawing on Foucault, she questions how social relationships between the patient and the clinic are changing, suggesting that not only is the status of the patient changing, but also the definition of the medical professional (Adams, 2017). Central to her analyses is the notion of surveillance. Drawing on post-panoptic theory, she has teased apart the multidirectionality of surveillance practices involved in online review and rating sites and, through the concepts of sousveillance, coveillance and infoveillance, shown how patients ‘are enrolled into participatory surveillance structures where they monitor professionals/institutions (and by extension, the state), each other's opinions (especially if actually using the site to make choices) and their own behaviour’ (Adams, 2013). Adams’ work usefully raises the question of who is watching whom and suggests an inversion of the medical gaze inasmuch as patients are involved in surveilling doctors. While Adams’ work provides a useful set of provocations about the ways in which digital surveillance is re-shaping the gaze, what remains unanalysed is both the subjectification entailed in changing knowledge practices and the extent to which the gaze extends not only to individual healthcare interactions but to the healthcare service writ large.

In this paper, we draw on an empirical study of GP practice staff to show how the gaze is turning, not simply from the patient to the healthcare provider, but *additionally to the body politic of the NHS*. While the notion of surveillance is implicit in our analysis, it is the *effects* of this surveillance with which we are concerned, namely the ways in which the discourses of online reviews and ratings produce new professional subjectivities among healthcare professionals. According to Foucault, knowledge makes people its subject, since people make sense of themselves in reference to the various bodies of knowledge that are available to them in a given time and place (Foucault, 1983). Discourse refers to ‘historically variable ways of specifying knowledge and truth—what it is possible to speak at a given moment’ (Ramazanoglu, 1993). The predominant discourse of online reviews and ratings in healthcare is that of patient empowerment (Johansson et al., 2021); however, a counter-discourse of healthcare worker *disempowerment* is evident at the fringes, summed up in a *British Medical Journal* piece (tellingly located in the ‘Careers’ section) entitled ‘You are being watched: panopticons in health care’ (Wessely & Gerada, 2016). In our data, we identify three counter-discourses characterising the evolving ways in which online reviews and ratings are creating new subjects in primary care practices: victimhood, prosumption versus traditional values and taking control. As we go on to show, the way in which staff speak about online feedback is patterned by the social environment in which they work and the constraints of the NHS they encounter on a day-to-day basis.

## Methods

We conducted a qualitative study involving interviews and focus groups with key practice staff, including general practitioners, practice nurses, managers and receptionists between May and September 2019. Practices were identified through the NIHR Clinical Research Network (CRN), who worked with the research team to identify a range of practices from more and less affluent areas and of differing profiles (size, number of staff and urban/rural). Practice managers and lead GPs were contacted by the CRN and invited to share information about the study with their colleagues before deciding whether they wanted to take part. A total of five practices agreed to take part.

Each practice was asked to identify a minimum of one GP, practice nurse, practice manager and receptionist to take part in an individual interview and a focus group to discuss their views of online patient feedback. All eligible staff were invited to participate. A total of 41 staff took part across the five practices; the data reported on in this paper consist of 22 individual semi-structured interviews and 5 focus groups, as detailed in Tables 1 and 2.

The aim of the focus groups was to understand more about the practice's approach to feedback and provide further knowledge than that collected in the interviews about staff members’ perceptions and experiences. During the discussion, participants were invited to discuss actual examples of online patient feedback posted on NHS Choices about their practice and how the practice had responded or would respond to this feedback. Across both data collection methods, we left the definition of ‘online feedback’ intentionally broad, covering everything from sanctioned feedback administered by the NHS, such as the Friends and Family Test and NHS Choices; third-party feedback sites such as Care Opinion and iWantGreatCare.com; search engine platforms such as Google reviews; and social media platforms such as Facebook and Twitter.

All interviews and focus groups were conducted by CM, recorded, transcribed and entered into NVivo 12 for analysis. Data were analysed iteratively following the constant comparative method. Memo-writing and interpretation followed Charmaz’ vision of a reflexive, constructivist grounded theory that digs deep into the empirical while building ‘analytic structures that reach up to the hypothetical’ (Charmaz, 2006). An initial process of detailed line-by-line coding within interviews led to the development of a set of provisional categories, used to code subsequent transcripts in a more focussed manner. This iterative process involved testing the adequacy of categories against the data (constantly turning between codes and data) and then of moving between cases (comparing data to data).

## Findings

Below, we organise our findings into three sections. Firstly, we describe a pervasive feature of the accounts offered by primary care staff in this study, namely an acute negative response to online patient feedback. In so doing, we are sensitised by Lupton's observation of the need for a critical analysis of digital technologies to pay attention to the affective and sensory dimensions of lived experience (Lupton, 2017). We then go on to present three counter-discourses to the prevailing ‘choice and empowerment’ discourse of online reviews, which staff in this study used to make sense of their day-to-day work of seeing patients. We show how these discourses are patterned by the social make-up of the GP practice and reflect concerns with under-resourcing, a shift to market values and the need to take control. Finally, we show how the nature and direction of the gaze are changing, from the bounded and private space of the clinic to the dispersed and ubiquitously visible domain of the online forum.

## ‘Techniques Of Feeling’ Digital Feedback

Within the sociological literature, there has been increasing attention to the embodied emotional work that healthcare staff engage in as part of their everyday encounters with patients (Litvina et al., 2020; Nettleton et al., 2008; Wong et al., 2018). So far, little has been said about the role of patient feedback in shaping these experiences and how online review and rating sites shape the ‘techniques of feeling’ (Nettleton et al., 2008) that primary care staff engage in. In policy documents, online patient feedback is presented in business-like terms, yet our interviews and focus groups showed it to be anything but a dispassionate tool for quality improvement. In common with previous research showing that staff predominantly experience such feedback negatively (Patel et al., 2015), the participants in this research almost universally spoke about the negative feelings online patient feedback elicited in them. This is in spite of evidence showing that most online feedback is positive (Boylan et al., 2019, 2020).

The staff we spoke to were candid about the way feedback made them feel and identified both a range of negative emotions (Table 3) and different levels on which these emotions worked.

Emotions covered everything from anger to humiliation, shame, fear and despondency, as the following extracts illustrate:

“It is a really hard job, and getting negative feedback - it makes you feel humiliated actually” (Practice 3, GP, interview)

“We don’t do sick. We will drag our arses in with coughs and colds feeling like crap, mentally emotionally and physically to serve the public that then moan about us. Yeah it's, it's pretty heart-breaking, really, and soul destroying.” (Practice 4, nurse, interview)

Staff relayed that online patient feedback could affect them very personally, or that they had known colleagues to suffer deeply as a result of comments patients left online. Some said that receiving such comments could lead them to practise defensively, while others suggested it could have consequences for filling vacant posts. One participant said they knew of a colleague who had decided not to apply for a position at one local practice because of the vitriolic patient feedback she had seen online.

“[Online comments] they’re really, they’re really personal. And, and it really affects you, you know, they affect people very badly I mean and, you know, in the back of your mind you practise differently and you respond different, it really hangs over people…some of them are really vitriolic and really unpleasant and it's really difficult to, to just kind of move on from it.” (Practice 1, GP, interview)

“I haven’t looked in the last year, I haven’t been brave enough. And that shows, I mean I literally would feel terrified to look because if there was something terrible about me online I don’t know what I’d do.” (Practice 3, GP, interview)

Talk about the negative emotional impact of online patient feedback occurred at several levels: personal, professional and public. On a personal level, practice staff were upset when they felt they had tried their best but the patient wasn’t happy. On a professional level, staff felt aggrieved that comments and ratings were used to subject the practice to government benchmarking in an unjust and non-transparent way. Finally, staff at some practices despaired at the public naming and shaming of the ‘worst’ practices for feedback in the press. These kinds of sensationalising stories did not take factors such as local demographics and resources into account.

“I feel it's unfair to use patient experience as a marker of how well we are doing in general practice and I feel sometimes quite demoralised by that.” (Practice 4, GP, interview)

While all kinds of staff at all of the practices expressed some degree of negativity about online patient feedback, there was a stark difference in the degree to which this occurred and the prevailing discourses around the data, as we go on to explore below.

## ‘Choice’ and ‘Empowerment’: Counter-Discourses

The degree of acuity with which practice staff experienced online patient feedback in negative emotional terms varied greatly between practices. We identified very different sets of counter-discourses about online patient feedback, which mapped to a large degree onto the different practices in our study. These were not always discrete, indeed they sometimes overlapped, and there was inevitable heterogeneity in the voices within practices.

### Victimhood

The most familiar discourse was that of victimhood. Central to this discourse was the sense of injustice occasioned by a perceived lack of the right to reply when patients left negative comments online. While patients could write whatever they wanted and could hide behind the cloak of anonymity, practice staff could not tell their side of the story for fear of jeopardising professionalism, patient confidentiality or both. Because staff felt they could not challenge the veracity or accuracy of accounts and had no space to provide their own version of events, they likened feedback sites to a one-way street. This is in contrast to the policy discourse, patient expectations and the design of patient feedback sites, which all prescribe that staff respond. Metaphors of violence were characteristic of this narrative—of staff being ‘clobbered’ by negative feedback or ‘getting a battering’, of patients ‘ganging up’ on the practice on social media, of it being ‘dangerous’, ‘sinister’ and a ‘weapon’. Indeed, some spoke of GPs being easy and ‘vulnerable targets’ for complaints against a complex and sometimes inadequate system that was not of their making.

“It just feels a very one sided kind of way of communicating … It feels like it's almost like they are able to put it out there without any right of reply.” (Practice 3, focus group)

“It seems like a very unilateral way because we’re never allowed to give our experience of the patients to anyone. You know…everything has to stay in these four walls and it's almost like people think we don’t have feelings or experiences ourselves.” (Practice 4, GP, interview)

“R: It's almost dangerous, isn’t it, that when they just put them out there like that, to me…

Facilitator: Tell us a bit more about that.

R: I just feel when they put these negative - that they are almost, it's almost like sinister when they are really, like [GP name] said, you are open to those comments and you think—

R: It does just make people gang up, doesn’t it…” (Practice 3, focus group)

“We don’t have a voice to come back against any of it” (Practice 3, GP, interview).

Systemic problems in the NHS as the root cause of negative feedback were a recurring trope. The victimhood narrative was most acute amongst those who felt aggrieved at the under-resourcing of the NHS and the resulting working conditions for NHS staff. Staff felt it was unfair to be the subject of negative patient feedback when the issues patients raised (such as waiting times to get a GP appointment) were beyond their control.

“I do totally get that in lots of ways we’ve got all the power, but actually resources where I work are so strapped, it's really hard when you are doing your absolute best to run the very best service you can under incredibly difficult circumstances when you don’t have enough staff or resources, to be told that you’d be running a better service if you -, you know it's not like we don’t know that.” (Practice 3, GP, interview)

“I think that asking for feedback all the time gives the patients the impression that we’ve got more capacity in general practice than we do have. So, I think it, it gives a false sense of expectation.” (Practice 4, Practice Manager, interview)

“This is the NHS. It's the NHS that's on its knees, up to its eyeballs in debt, has no money to do anything with. And do I find rating us a good thing? Do I hell as like, no. We’re doing the best with what we’ve got. Give us a huge injection of money, massive injection of billions and then come back to us and then tell us that we’re good… But when we’re on our knees? Don’t. And people know the NHS is on its knees and how it is, so don’t sit there slagging and slating, yeah? No, no I can’t bear it. So no, we shouldn’t be tarred with this whole stars-, nobody should, because everybody's just trying to do their best in life.” (Practice 4, nurse, interview)

As can be seen, the quotes above all come from Practices 3 and 4. Practice 3 was in a relatively deprived area, with high levels of ethnic diversity and non-English speaking patients; Practice 4 had a legacy of management problems and had recently gone through a merger involving a large increase in patient numbers. Whereas the other practices in the study batted away the victimhood discourse, these two practices narrated online patient experience data as an attack, betraying a sense of vulnerability and lack of control. We suggest that the victimhood discourse allowed staff to make sense of providing care to patients in a context in which they felt otherwise disempowered—by structural constraints, management problems and challenging working conditions. By contrast, those practices that felt confident in themselves, well resourced, and which had a patient population that was young, mostly white and affluent were more adept at recounting resilient narratives, as we explore below.

### Prosumption versus traditional values

Prosumption is a term used to describe the simultaneous production and consumption of digital content in the Web 2.0 era (Beer & Burrows, 2010; Lupton, 2014). Familiar through ratings and review sites for commercial enterprises—Tripadvisor being the archetypal example—the parallels in the digital health domain were readily made by participants in this study, who rejected the re-articulation of healthcare practice as a form of service work. In the ‘Prosumption vs Traditional Values’ discourse, participants contrasted the values of healthcare in the NHS with a capitalist logic of choice and competition underpinning the commercial enterprises which commonly make use of consumer feedback. For a variety of reasons, the latter was not felt to be an appropriate basis for evaluating healthcare quality in primary care. Firstly, presenting patient feedback sites as a means to help patients choose a practice was felt to be irrelevant because there was, in effect, no choice to be had:

“We are a practice that largely speaking is the only practice that patients can register with, because there is some overlap with adjacent practices, but not a great deal here. So there is no great competition here - that to promote customer service - and whilst I’m sure all of us would like our patients to leave happy, actually, most of us want them to leave healthy. It doesn’t always equate to the same thing. So the feedback from that point of view doesn’t necessarily lead you to where you want to be.” (Practice 5, GP, focus group)

“[I]f it does mean that you know you’ll come to the top of the, ‘Which doctor should I register with in [city name]?’ Well does that matter? Possibly, I don’t know. We seem to have enough patients and potentially there are going to be more patients to go round as more practices go to the wall, as is increasingly happening…some parts of the country have no GPs at all…So, what's the point of patient feedback when there are no doctors to complain or praise?” (Practice 2, GP, interview)

R: I do think that whilst a GP practice is a business, it's not a business in the same way that a rail company is or—I think sometimes it's not helpful to encourage patients—

R: It was never a choice to make it a business either.

R: I don’t think it's helpful for patients to think of it in exactly the same way as they think of the bus company and the cinema and the—

R: There is quite a pressure, potentially politically, to move it that way. (Practice 2, focus group)

Secondly, feedback sites were said to conflate good medicine with good patient feedback, when in fact, the inverse relationship could be observed. That is, participants felt that patients who ‘did not get what they wanted’—be it antibiotics, stronger opiates, or some other course of action—were inclined to leave negative reviews even though the right clinical decision had been made. One participant backed this up referencing a paper by Ashworth et al. (2016), demonstrating that GP practices that prescribe fewer antibiotics have lower patient satisfaction scores on the General Practice Patient Survey (GPPS). Another summed it up as follows: ‘we are not really here to make people happy. We are here to make them well’ (Practice 5, focus group), again rejecting the notion of healthcare practice as service work.

Finally, the staff at one practice in particular (Practice 2) propounded a strong counter-narrative to prosumption in the form of ‘traditional values’. Practice 2 was a small, long-established practice occupying a historic building in an affluent city-centre location. The practice staff prided themselves on their strong inter-personal relationships with their patients and their face-to-face style of communication. The best feedback was said to occur in a weekly walking group that one of the GPs undertook with patients, and to be evidenced through gifts brought in both regularly and at Christmas, when ‘we could fill the carpet in reception if you were to lay out gifts’ (receptionist).

“R: Where do people go if they don’t go online mostly? Most people know us and they come in and see us. They bring baklava or they bring banana cake. They come in and they bring personal thanks, personal tributes or they come in and thank you the next time. If they’ve got a complaint then they come in and have a moan at the desk or if they want to, they write something down.

R: That's a sign of a very good practice-patient relationship essentially, whether they’ve got the confidence to come and have a moan and even mention our names and things” (Practice 2, focus group)

“I hate to use the word cosy, but I think that might refer to us a little bit as a practice. And the online thing comes a little bit outside of that, it's a bit all modern world isn’t it?” (Practice 2, receptionist, interview)

While the ‘Prosumption vs Traditional Values’ discourse was largely negative about online patient feedback, in contrast to the victimhood discourse, it came from a place of security and was, therefore, dismissive rather than fearful. In rejecting the premise of digital interactions about patients’ experiences, and thereby not engaging with online feedback, staff retained control over practice identity and their own account of what good care looked like. This involved re-inscribing the work of surgery staff as medical practice rather than service work.

### Taking control

If the discourse above was about maintaining control in the face of undesirable modernising tendencies, a third discourse, ‘Taking control’, provided a more positive and entrepreneurial engagement with the online patient experience landscape. Within this, online feedback was framed as ‘a force for good’, providing transparency, accountability and widening access for patients.

“[Y]ou can actually look at it as a very useful service that patients provide to the NHS” (Practice 1, GP, interview)

“It gives other people a chance to think about the comment and possibly add something to it. Also, you can’t hide things under the carpet can you? You know, if it's there and there has been a complaint or a positive comment, then you’ve got to do something about it really, haven’t you? You’ve got to take notice of it. You can’t just ignore it…and think, ‘Oh well no-one else is going to find out about that.’” (Practice 5, nurse, interview)

While many of the sites told of ‘horror stories’ in their early experiences engaging with the online world, two sites in particular transformed these into a discourse of proactively taking control. This involved measures such as shutting down bogus sites; directing patients to leave feedback on legitimate platforms, such as NHS Choices; creating a practice Facebook page to communicate with patients; developing a social media strategy; and employing a patient services manager with responsibility for managing patient feedback. Characteristic of this discourse was a sense of agency in responding to online feedback (e.g. through the functionality provided by sites like NHS Choices) rather than the sense that there was no right to reply. Staff made upbeat and optimistic comments about being responsive, proactive, in control, and future-oriented.

## A Shifting Gaze

So far, we have shown that rather than being a dispassionate management tool for quality improvement, online patient feedback elicits strong emotional responses in practice staff. In describing the counter-discourses above, we have shown that rather than seeing online patient feedback as a straightforward representation of patients’ experiences of receiving care, GP practice staff mobilise it as a way to talk about and make sense of the conditions in which they work. This helps to explain the paradox that while most online patient feedback is positive, it is often perceived to be negative. However, as the extracts above also indicate, online patient feedback is fundamentally changing the doctor–patient relationship by providing a public forum for what would previously have been known only within the confines of the doctor's surgery. That is, the nature of the gaze is changing; in addition to speaking for the otherwise silent pathology within their own body, the patient now also speaks for the otherwise silent pathology within the body politic of the NHS. Whereas the former—the clinical gaze—depended on the specificities of the clinical space (Foucault, 2012), the latter is characterised by public visibility and wider attention.

Staff in this study were attuned to this changing social relation and articulated it by reference to the visibility of different forms of digital and material communication. Perhaps counter-intuitively, material forms of feedback, such as thank you cards, chocolate and cake, were perceived to be invisible to others, whereas digital feedback in the form of reviews and ratings was perceived to be highly visible to all:

“A good feedback can come over the front desk. Patients leave cards and like presents or whatever and it's, yeah. That is not out there for everybody to see that we’ve had, you know, something really positive back.” (Practice 1, focus group)

“If they complain on a social media site and just put a blank, ‘it was a terrible service, blah, blah, blah’ then I think that's much more difficult to deal with than something that comes to the surgery and no-one else sees. It's very difficult to comment on…and it's seen by a lot of people.” (Practice 5, focus group)

Not only that, but whereas physical expressions of feedback were said to maintain professional distance between staff and patients, online feedback was felt to be more personal. Although staff did not explicitly refer to hierarchy or power, we might deduce that this is also implied in references to a loss of professional distance, as below:

“You know, so you have to take yourself out of the equation and I do think the professionalism of medicine is a little bit about having that distance so that you don’t take it too much to heart and that's what possibly online weirdly reduces that distance” (Practice 2, GP, interview)

The prerogative to determine the terms of communication was taken away by online platforms—whether social media or formal data collection platforms such as Care Opinion and NHS Choices:

“I’m perfectly happy for my clinical decisions to be challenged, in fact I kind of often welcome it, usually it's the other person's right. But to be able to do anything useful about it I need it to come to me in a form that can start a dialogue” (Practice 3, GP, interview)

“I know the partners were really low that they got that sort of feedback from this person, so, yeah, I think because it was more social media that that was put out on, of course it drew more and more people into it and it got out of hand and it, it escalated and it shouldn’t have done.” (Practice 3, nurse, interview)

In the second quote, we see how social media amplifies what once would have been a personal communication between doctor and patient, and the ensuing sense of disempowerment that was experienced. Attempts to regain control of the terms of engagement were made by those practices voicing the more entrepreneurial discourses above, for example, by creating a practice Facebook page/Twitter account and by proactively directing patients to these practice-curated spaces.

## Discussion

In this paper, we have drawn on an analysis of how NHS primary care staff experience online patient feedback to show that: (1) online patient experience data elicit strong emotional responses in practice staff, (2) staff from different practices make sense of these data in different ways, propounding discourses which help make sense of their working conditions and practice identity, (3) online patient feedback troubles the clinical gaze by transcending the space of the clinic through its digital reach. This has implications both for what is said by the patient and what is heard by the practitioner. Rather than speaking only for the silent pathology inside their body, the patient now also speaks for the silent pathology inside the body politic. That is, the issues perceived to be most commonly raised—waiting times to get an appointment, triaging by reception staff, problems with prescriptions—are indicative of a system straining from years of underinvestment, staff recruitment challenges and increasing workload demands (Madan et al., 2017; Owen et al., 2019). Our analysis helps understand how broader social relations are being forged in an NHS requiring significant increases in investment (Anderson et al., 2021; Baird et al., 2016) and progressively subject to benchmarking based on patient experience metrics (Duschinsky & Paddison, 2018). In this context, reports that GP practice staff are burned out and the profession in crisis are not uncommon (Cheshire et al., 2017; Riley et al., 2018; Salisbury, 2019).

A general sense of concern about online patient feedback was pervasive across all practices. This may be a response to the marketisation of healthcare (Gabe et al., 2015) and a reaction against the perceived mischaracterisation of medical practice as service work. It may also reflect the broader cultural context in which online reviews are perceived to be a tool to berate businesses for poor service. However, the degrees of concern varied in our dataset, with online patient feedback eliciting an acute and negative reaction at some sites more than others. For some in our sample, online patient feedback represents an opportunity to take control, or is simply not seen as part of their identity, while for others, it represents a threat or an injustice. The ‘silent pathology’ affecting a practice may be deprivation in the local community and the attendant difficulties of providing good service with limited means; for others, it may be organisational problems and politics, such as a practice merger or a legacy of poor management leading to current problems.

While online review and rating sites, then, are undoubtedly a form of surveillance, it is important that we remain attentive to the knowledge/power relations and subjectification that are its effects—not only for patients, but also for healthcare providers. Just as the disciplining effects of wearable technologies, self-tracking devices and online review and rating sites on patients have been noted (Lupton, 2014; Petrakaki et al., 2018; Sharon, 2017), so too should we note the objectification and normalisation of the disciplined healthcare provider. As online patient feedback platforms gain prominence and policy clout in primary care, the potential of these sites to create self-regulating ‘docile bodies’ within the workforce of the NHS should remain a question of interest. As our findings show, there are a range of counter-discourses through which healthcare providers are resisting and refashioning the techno-utopian empowerment imperative of digital patient feedback. However, in practices in areas of greater social disadvantage and/or with a history of organisational upheaval, the victimhood discourse—coupled with increasing work pressures and declining GP numbers (Iacobucci, 2019)—should be a cause for concern.

## Conclusion

Over 20 years ago, Armstrong wrote, ‘Doctors will have to learn that a satisfied patient is as important as a medically improved one’ (Armstrong, 1990). In this paper, we have charted the emotional work that primary care staff undertake as this learning takes shape in the digitally surveilled clinic. As the gaze turns from the pathophysiology of the patient to the space of the clinical interaction itself, the patient's view takes on new salience, engendering relations of power given ballast by the promise of choice, efficiency and personalised care. Within this sphere, those primary care staff who embrace the digital, solicit online feedback on their own terms and take control of the patient experience narrative may find the power shifts in their favour. In those practices that struggle to meet expectations grounded in a marketised approach to healthcare and based in part on patient experience metrics, staff will continue to hear the patient's voice as a symptom of the silent pathology of the NHS.

## Figures and Tables

**Figure 1:**
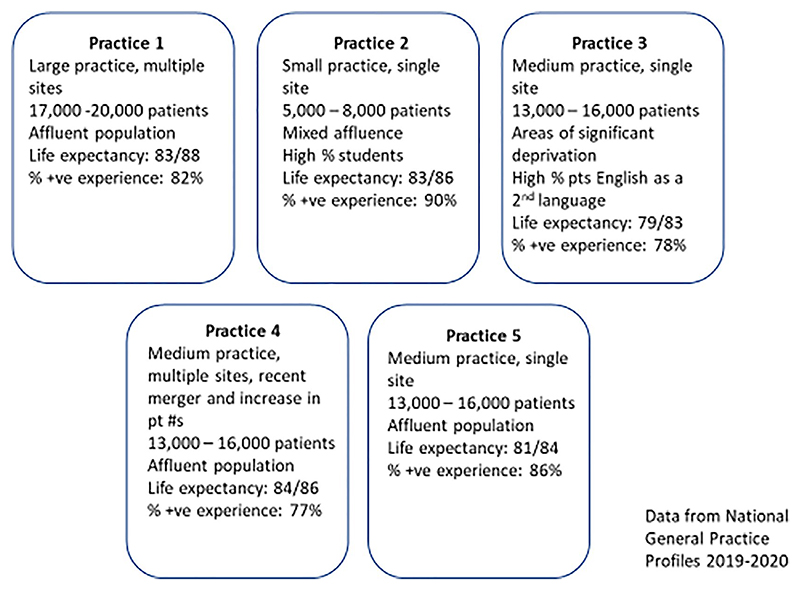
Profile of participating practices

**Table 1:** Interviews conducted

	Interviews *(n* = 22)					
	GPs	Receptionists	Managers	Nurses	Total	
Site 1	1	2	2	1	6	
Site 2	1	2	1	1	5	
Site 3	1	1	1	1	4	
Site 4	1	1	1	1	4	
Site 5	1	0	1	1	3	
Total	5	6	6	5	22	

**Table 2:** Focus groups conducted

	Focus groups *(n* = 5)					
	GPs	Receptionists	Managers	Nurses	Total	
Site 1	1	1	1	1	4	
Site 2	3	2	1	1	7	
Site 3	3	3	2	4	12	
Site 4	1	1	1	1	4	
Site 5	6	1	1	2	10	
Total	14	8	6	9	37	

**Table 3:** Coding for emotional responses to online patient feedback

Negative emotions	Positive and neutral emotions
Demoralising	Feel appreciated
Dread, makes you feel sick, scared to look at it	Gives you a boost, good for morale
Feel angry	
Feel defensive	Grateful
Feel despondent and disengaged	Don’t take it personally
Feel exposed and vulnerable in public	
Feel humiliated	
Feel named and shamed	
Feel threatened or attacked	
Feel unable to respond or defend yourself	
Frustrating	
Hurtful	
Makes you feel rubbish	
Shock	
Take it personally	
Upsetting	
Worry about reputation	
